# Effects of Acorns on Subcutaneous Fat Deposition in Yuxi Black Pigs by Transcriptomic Analysis

**DOI:** 10.3390/metabo15020071

**Published:** 2025-01-23

**Authors:** Zhe Sun, Dongyang Liu, Siyuan An, Jinzhou Zhang, Lei Lei, Zhiguo Miao

**Affiliations:** 1 College of Animal Science and Veterinary Medicine, Henan Institute of Science and Technology, Xinxiang 453003, China; sz18437911393@163.com (Z.S.); liudy97@126.com (D.L.); asy15903076062@163.com (S.A.); zhangjz69@126.com (J.Z.); 2 Zhengzhou Agricultural Comprehensive Administrative Law Enforcement Detachment, Zhengzhou 450044, China

**Keywords:** acorns, Yuxi black pig, lipid deposition, transcriptome sequencing

## Abstract

**Background/Objectives:** The backfat thickness of pigs is closely related to dorsal subcutaneous fat deposition and meat quality, and appropriate reduction in backfat thickness is important for improving pork quality. The present study investigated the effect of acorn diet on the backfat thickness and lipase activity of Yuxi black pigs and to gain further insight into the molecular mechanism of the acorn diet on the dorsal subcutaneous fat deposition of Yuxi black pigs by transcriptome sequencing (RNA-seq). **Methods:** Thirty-six Yuxi black pigs with an initial body weight of 99.60 ± 2.32 kg (three replicates per group and six pigs per replicate) were randomly divided into two groups (CON group was fed a basic diet and AEG group was fed 30% acorn diets). Pigs were individually fed twice daily and had access to water ad libitum throughout the experiment. The test period was 4 months. **Results:** Results showed that backfat thickness and ACC, MDH, and LPL lipase activities were significantly reduced in the AEG group than in the CON group (*p* < 0.05). In addition, RNA-seq identified 826 differentially expressed genes (DEGs), with 505 up-regulated and 321 down-regulated. The DEGs were significantly enriched in the lipid metabolism process and lipid catabolic process, fatty acid (FA) catabolic process, and FA β-oxidation according to GO enrichment analysis. *LEP*, *CHPT1*, *UCP3*, *ACOX1*, *SCD5*, and *ACAA1* were screened as key differential genes regulating dorsal subcutaneous fat deposition. **Conclusions:** The above results indicated that feeding the 30% acorn diet could regulate the expression of genes involved in fat deposition and reduce lipase activity, thereby decreasing the backfat thickness, inhibiting the deposition of dorsal subcutaneous fat, and improving the pork quality. The findings of this experiment established a basis for subsequent research into the molecular mechanisms underlying the impact of acorn diets on fat deposition in Yuxi black pigs and provided the scientific evidence to promote the exploitation and industrialization of acorns.

## 1. Introduction

Pork provides the vast majority of protein to consumers around the world, contributing greatly to a balanced human diet and good health. However, as the standard of living of people in developed and newly developing countries improves, so do the health requirements for animal food, and the pork consumed is required to have a high health index. Fat is one of the main factors affecting the quality and health index of pork. Fat can affect the flavor, tenderness, juiciness, and fatty acid composition of pork, and the content and proportion of saturated and unsaturated fatty acids in fat determine the health index of pork [1]. Fat deposition is influenced by nutrient level, breed, and age [2]. Meanwhile, backfat thickness can reflect the fattening effect and fat deposition ability of pigs; this parameter is of significant importance in reflecting the deposition of dorsal subcutaneous fat deposition [3]. However, excessive fat deposition can reduce the nutritional value and economic value of pork. Wang suggested that feeding a betaine diet could reduce backfat thickness, inhibit subcutaneous fat deposition, and improve pork quality in Ningxiang pigs [4]. Wiegand found that adding conjugated linoleic acid to the diet could reduce subcutaneous fat and backfat thickness and improve pork quality [5]. The above results showed that it is important to reduce the subcutaneous fat deposition to improve the pork quality.

Acorns are wild plants widely distributed in the mountains of western China. They are rich in starch, protein, fat, amino acids, unsaturated fatty acids, and trace elements. At the same time, acorns have anti-diarrhea, hypoglycemic, and antioxidant effects and have been widely used in food additives [6]. It was found that feeding acorn diets can increase monounsaturated fatty acids (MUFA), reduce atherosclerosis, thrombosis, and peroxidized fatty acid nutritional indices of pork, and have a positive effect on pork quality and nutritional value [7,8]. Therefore, the utilization of acorns as a novel feed resource with considerable development and utilization value in pig production represents a promising avenue for further investigation. However, acorns are rich in anti-nutrient factor tannins, and excessive intake can inhibit the intestinal absorption of nutrients, thereby reducing the carcass quality and production performance of pigs [9]. Yuxi black pigs are a highly excellent local breed that is predominantly cultivated in Lushi County and Luanchuan County in the west of Henan Province. Their physical characteristics are mainly characterized by a black coat, large face, short mouth, thick and short neck, and strong limbs. Meanwhile, Yuxi black pigs have the characteristics of excellent meat quality, tolerance to rough feeding, early sexual maturity, and strong immune ability and their delicious meat is loved by local consumers [10].

RNA-seq (RNA sequencing technology) is a method that enables the rapid and comprehensive collection of transcriptional information pertaining to biological samples. This is accomplished through the comprehensive examination of cDNA sequences via high-throughput sequencing technology. Meanwhile, it is possible to study the function and regulatory mechanisms of genes at a holistic level and to mine functionally relevant candidate genes with RNA-seq. RNA-seq is now widely used in investigating fat deposition in pigs and has succeeded in identifying a number of key genes that regulate lipid metabolism [11,12]. However, the RNA-seq of an acorn diet on the subcutaneous fat of pigs is less studied. Our previous study found that 30% acorn diets can enhance the intramuscular fat (IMF) content and the quality of meat in Yuxi black pigs [13]. Therefore, the present study investigated the impact of a 30% acorn diet on the backfat thickness and lipase activity of Yuxi black pigs and identified the key genes involved in regulation by performing RNA-seq on dorsal subcutaneous fat. The findings of this experiment provide a basis for subsequent research into the molecular mechanisms underlying the impact of an acorn diet on fat deposition in Yuxi black pigs.

## 2. Materials and Methods

### 2.1. Experimental Design and Diets

The protocol for the tests was approved by the Animal Care and Use Committee process of Henan Institute of Science and Technology. A total of 36 healthy Yuxi black pigs with an initial body weight of 99.60 ± 2.32 kg (3 replicates per group and 6 pigs per replicate) were randomly divided into two groups (CON and AEG groups). The CON group was fed a basic diet, and the AEG group was fed a 30% acorn diet. Pigs were individually fed twice daily and had access to water ad libitum throughout the experiment. The test period was 4 months. The dietary formulations employed in this research were constructed in accordance with the nutritional guidance for the fattening of pigs as set forth by the National Research Council (NRC 2012). The composition of the diets for each group is presented in Table 1. An average of 2.5 kg of experimental diet was consumed daily during the trial to control body weight.

Composition per kg of premix: VA: 120,000 IU; VD_3_: 45,000 IU; VE: 700 IU; VK_3_: 45 mg; VB_2_: 150 mg; VB_6_: 50 mg; niacinamide: 750 mg; calcium pantothenate: 460 mg; choline chloride: 3.5 mg; Cu: 0.31 g; Fe: 3.5 g; Zn: 1.4 g; Mn: 0.8 g; I: 42 mg; Se: 7.8 mg; Ca: 16%; P: 3.5%. CON: basal diet; AEG: 30% acorn diet.

### 2.2. Slaughter and Sample Collection

When the average body weight of the pigs reached 140 kg, they were weighed following a 24 h fasting period. Subsequently, the backfat thickness of the experimental pigs was determined and the experimental samples were collected by electric shock, bleeding, and slaughter. The dorsal subcutaneous fat samples were extracted and rapidly stored in liquid nitrogen in 1.5 mL EP tubes for lipase activity detection and RNA-seq. After collection, all samples were transported to an ultra-low-temperature freezer at −80 °C.

### 2.3. Backfat Thickness

When the average weight of the test pigs reached approximately 140 kg, after bleeding and slaughtering, the average value of backfat thickness was determined using calipers at three points in the region of the first rib, the last rib, and the lumbar joint of the right carcass.

### 2.4. Determination of Lipase Activity

The concentrations of acetyl-CoA carboxylase (ACC), malate dehydrogenase (MDH), and lipoprotein lipase (LPL) were determined by enzyme-linked immunosorbent assay kit. Nanjing Jiancheng Bioengineering Institute (Nanjing, China) provided the kits.

### 2.5. RNA Extraction, Sequencing, and Transcriptome Data Analysis

Total RNA was extracted from dorsal subcutaneous fat using TRIzol reagent (Invitrogen, Carlsbad, CA, USA). RNA was quantified using spectrophotometry analysis (IMPLEN, Westlake Village, CA, USA). A260/280 ratios were observed to be within the range of 1.9 to 2.1. The extracted RNA (RNA integrity number (RIN) ≥ 7.0) was employed in the construction of sequencing libraries.

According to the manufacturer’s instructions (Illumina, San Diego, CA, USA), RNA purification, reverse transcription, library construction, and sequencing were executed at Shanghai Majorbio Bio-pharm Biotechnology Co., Ltd. (Shanghai, China). The construction of the RNA-seq transcriptome library was conducted following the instructions provided by the Illumina^®^ Stranded mRNA Prep, Ligation, utilizing 1 μg of extracted RNA. The Illumina sequencing platform was employed for the sequencing of RNA-seq sequencing libraries (Nova Seq 6000 Illumina).

Raw data were subjected to filtration using the fastq v0.23.4 and the high-quality sequences obtained following filtration were used for alignment to the Sus scrofa reference genome (https://ftp.ncbi.nlm.nih.gov/genomes/all/GCF/000/003/025/GCF_000003025.6_Sscrofa11.1, accessed on 1 December 2024) using the HISAT2 (version 2.0) software package.

### 2.6. Differential Expression and Functional Analysis

The identification of differentially expressed genes (DEGs) between the two groups was conducted using the DESeq2 package (version 1.12.4). We defined thresholds of fold change ≥ 2 and FDR < 0.05. Moreover, GO and KEGG functional enrichment analyses of DEGs were conducted utilizing Diamond (version 2.1.6) and KOBAS3.0 to ascertain in which metabolic pathways DEGs were demonstrably enriched. A *p* < 0.05 is generally considered to be remarkably enriched for GO terms and KEGG pathways.

### 2.7. The qRT-PCR Validation

Gene expression levels were quantified using qRT-PCR. Six genes that exhibited differential expression were randomly selected for validation (see Table 2). Total RNA was isolated utilizing TRIzol reagent from dorsal subcutaneous fat in Yuxi black pigs (Invitrogen, Carlsbad, CA, USA). RNA was quantified using spectrophotometry analysis (IMPLEN, Westlake Village, California, USA). The total RNA (approximately 1 μg) of each specimen was employed in the synthesis of complementary DNA via the PrimeScript™ RT Reagent Kit (Takara Bio Inc., Tokyo, Japan). SYBR Green RT-PCR Kit (TaKaRa Biotechnology, Dalian, China) and ViiATM 7 real-time PCR System were used for RT-PCR. The alterations in gene expression were evaluated employing the 2^–ΔΔCT^ methodology.

### 2.8. Statistical Analysis

The data are displayed with the mean values and their standard deviations and were analyzed using *t*-test with the SPSS 26.0 software. Data visualization was performed via Prism 6 software. Significance was defined at *p* < 0.05.

## 3. Results

### 3.1. Backfat Thickness

**Figure 1:**
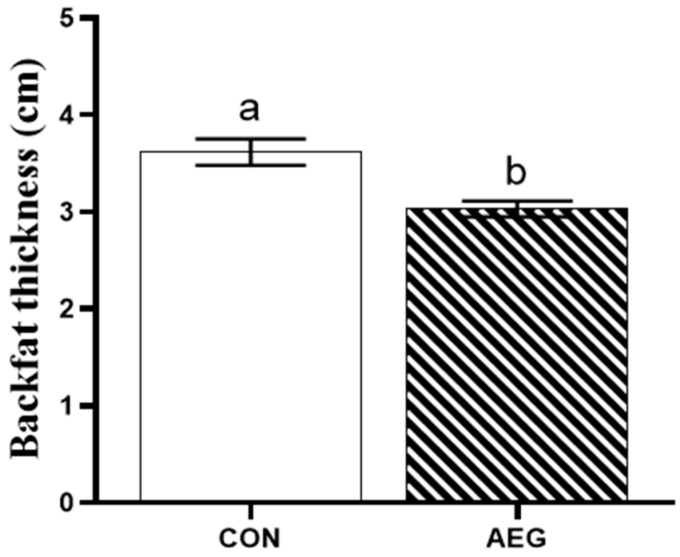
Effects of 30% acorn diet on backfat thickness. CON: basal diet; AEG: 30% acorn diet. The use of superscripts marked with different letters serves to highlight the existence of notable disparities between the two groups (*p* < 0.05).

Figure 1 shows that the backfat thickness of the AEG group fed the 30% acorn diet was observably decreased compared to the CON group (*p* < 0.05).

### 3.2. Lipase Activity

**Figure 2:**
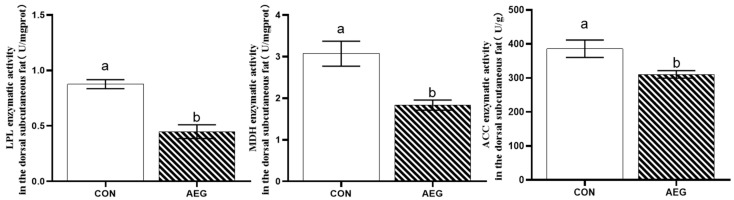
Effects of 30% acorn diet on lipase activity in dorsal subcutaneous fat of Yuxi black pigs. LPL, lipoprotein lipase; MDH, malate dehydrogenase; ACC, acetyl CoA carboxylase. CON: basal diet; AEG: 30% acorn diet. The use of superscripts marked with different letters serves to highlight the existence of notable disparities between the two groups (*p* < 0.05).

Figure 2 shows that LPL, MDH, and ACC lipase activities in the dorsal subcutaneous fat of the pigs fed the 30% acorn diet in the AEG group were found to be considerably decreased in comparison to the CON group (*p* < 0.05).

### 3.3. Overview of Sequencing Data

Transcriptome sequencing was performed on a total of six libraries, as shown in Table 3. After filtering the raw data, 44,888,886, 45,363,754, 46,014,420, 41,436,780, 47,571,278, and 53,530,398 clean reads were obtained from samples BZ-CON1, BZ-CON2, BZ-CON3, BZ-AEG1, BZ-AEG2, and BZ-AEG3, respectively. Useful reads of dorsal subcutaneous fat were 98.01–98.16% in the control group and 97.97–98.22% in the AEG group. The useful bases of the control group were 97.27–97.58%, and those of the AEG group were 97.22–97.72. In addition, the Q30 range of all samples was 94.94–95.31%, which was higher than 90%. This finding suggested that the sequence quality was sufficient to be utilized in subsequent analysis.

Table 4 shows that mapped reads of all samples were 97.63–97.96%. The multiple mapped values were 2.38–2.62%. Uniquely mapped were 97.38–97.62%. In addition, the exon mapped were in the range of 84.95–88.57%, while intergene mapped was 2.71–4.93%. The results showed that the reference genome had a good alignment with the sequencing results, the chosen reference genome was deemed appropriate for the purposes of this study, and the data were deemed to be valid.

### 3.4. Screening DEGs

**Figure 3:**
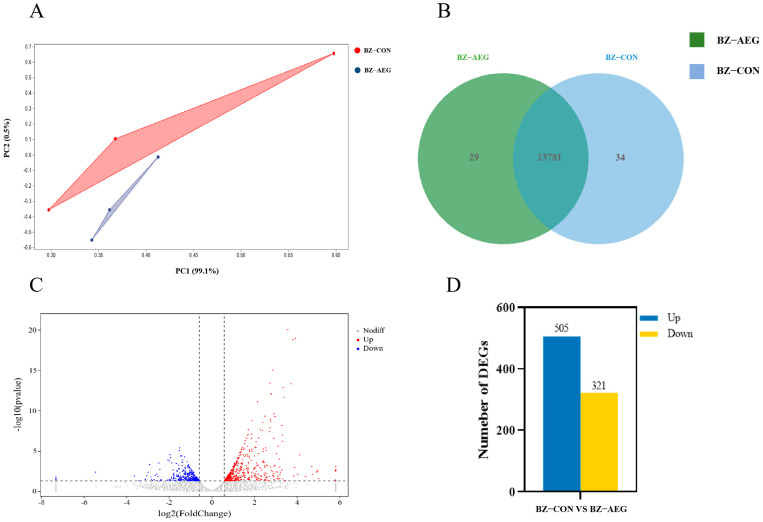
Identification of DEGs. (**A**) Sample principal component analysis diagram. BZ-CON was the dorsal subcutaneous fat of the control group; BZ-B was the dorsal subcutaneous fat of the AEG group. (**B**) Venn diagram of dorsal subcutaneous fat CON vs. AEG groups. BZ-CON was the dorsal subcutaneous fat of the control group; BZ-B was the dorsal subcutaneous fat of the AEG group. (**C**) Differential genes’ volcano map of dorsal subcutaneous fat. The gene with *p* < 0.05 and log2 fold change > 0.58 is marked in red; the gene with *p* < 0.05 and log2 fold change < −0.58 is marked in blue. (**D**) The statistical analysis of DEGs between CON and AEG groups. BZ-CON was the dorsal subcutaneous fat of the control group; BZ-AEG was the dorsal subcutaneous fat of the AEG group.

As can be seen from Figure 3A, PCA (principal component analysis) was performed on each sample according to the expression level, and the results showed that the samples had good repeatability. In Figure 3B, 13,781 genes were expressed in the dorsal subcutaneous fat between the CON and AEG groups. Figure 3C provides a visual representation of the volcanic map of differential genes in the dorsal subcutaneous fat. According to Figure 3D, 826 DEGs were identified in the dorsal subcutaneous fat, with 505 up-regulated and 321 down-regulated.

### 3.5. GO Enrichment Analysis of DEGs

**Figure 4:**
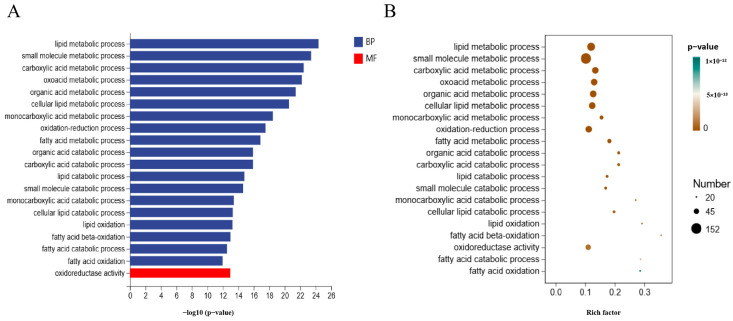
GO enrichment analysis of DEGs. (**A**) Bar graph of GO enrichment pathways for DEGs in dorsal subcutaneous fat between CON and AEG. (**B**) Factor map of GO enrichment pathways for DEGs in dorsal subcutaneous fat between CON and AEG.

GO enrichment analysis was conducted for 826 bases of DEGs in the dorsal subcutaneous fat control group and AEG group, and the enrichment pathways of TOP20 are shown in Figure 4A,B. Pathways related to lipid metabolism mainly include cellular lipid metabolic process, lipid metabolic process, lipid oxidation, fatty acid metabolic process, fatty acid catabolic process, cellular lipid catabolic process, fatty acid beta-oxidation, lipid catabolic process, and fatty acid oxidation.

### 3.6. KEGG Pathway Analysis of DEGs

**Figure 5:**
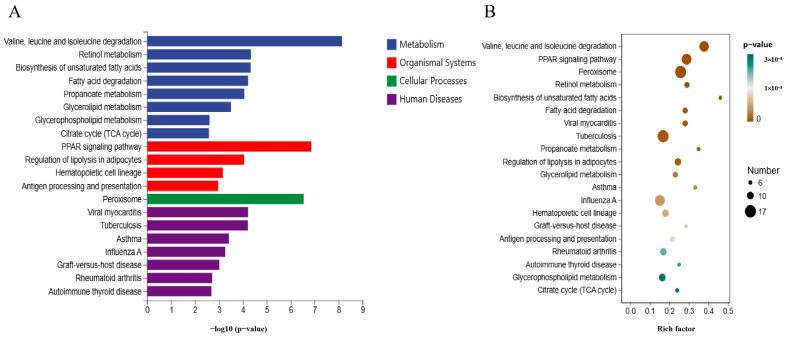
KEGG enrichment analysis of DEGs. (**A**) Bar graph of KEGG enrichment pathways for DEGs in dorsal subcutaneous fat between CON and AEG. (**B**) Factor map of KEGG enrichment pathways for DEGs in dorsal subcutaneous fat between CON and AEG.

KEGG pathway analysis was conducted on 826 bases of DEGs in the dorsal subcutaneous fat control group and AEG group, and the enrichment pathways of the TOP20 are shown in Figure 5A,B. Pathways related to lipid metabolism mainly include fatty acid degradation; biosynthesis of unsaturated fatty acids; propanoate metabolism; glycerolipid metabolism; valine, leucine, and isoleucine degradation; glycerophospholipid metabolism; citrate cycle (TCA cycle); PPAR signaling pathway; and regulation of lipolysis in adipocytes. Ten genes, including *LEP*, *CHPT1*, *UCP3*, *ACOX1*, *SCD5,* and *ACAA1*, were identified as key differential genes influencing lipid metabolism (Table 5).

### 3.7. The qRT-PCR Validation of Transcriptome Data Results

**Figure 6:**
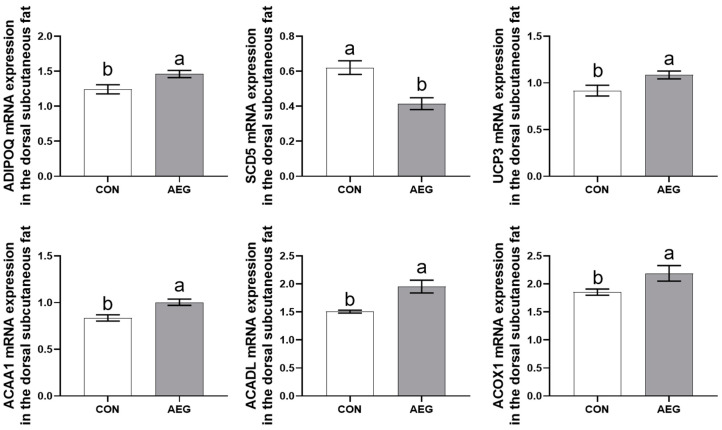
The qRT-PCR validation of the DEGs analyzed by RNA-seq. CON: basal diet; AEG: 30% acorn diet. The use of superscripts marked with different letters serves to highlight the existence of notable disparities between the two groups (*p* < 0.05).

ADIPOQ, SCD5, UCP3, ACAA1, ACADL, and ACOX1 in DEGs were chosen for verification of the RNA sequencing results using qRT-PCR, as illustrated in Figure 6. Data obtained by selected DEGs in RNA-seq were found to be in accordance with those obtained through RT-PCR. The results presented herein serve to substantiate the veracity of our sequencing and analysis methodologies.

## 4. Discussion

### 4.1. Backfat Thickness

As living standards have improved, there has been a concomitant change in consumer demand for pork, with a shift from a focus on quantity to one on quality. In pig breeding, subcutaneous fat content can directly affect pork quality and economic value. Subcutaneous fat is the main part of fat deposition in animals, and backfat thickness is not only the result of dorsal subcutaneous fat deposition but also the direct reflection of fat deposition ability [14]. One of the key objectives in improving pork quality is to increase the percentage of lean meat by reducing backfat thickness, which can be considered the main predictor of lean meat yield [15]. It can be reasonably argued that an in-depth examination of the regulatory mechanisms governing backfat thickness and subcutaneous fat deposition in Yuxi black pigs fed the acorn diet to improve pork quality is of considerable importance. The findings of this study indicated that the backfat thickness of the AEG group fed the 30% acorn diet was observably decreased compared to the CON group. He suggested that dietary glycine supplementation can prominently reduce the backfat thickness of pigs, consequently enhancing the quality of pork [16]. A further study demonstrated that the inclusion of varying levels of green tea by-products in the diet of pigs resulted in a notable reduction in backfat thickness and an increase in lean meat percentage and meat quality [17]. The findings of this study are in alignment with the aforementioned results: feeding an acorn diet could reduce backfat thickness and improve pork quality in Yuxi black pigs. This may be because acorns are wild plants rich in polyphenols, and phenolic compounds in them play an important role in inhibiting subcutaneous fat deposition [18]. Other studies found that there was no significant difference in backfat thickness between free-range pigs fed acorn and grass and captive pigs fed a standard diet but the activities of lipogenic enzymes were decreased, indicating that acorn feeding had an inhibitory action on subcutaneous fat deposition [19]. The discrepancy between the current findings and those of the preceding study may be attributed to variations in feeding systems, breeds of pigs, and acorn sources.

### 4.2. Lipase Activity

Subcutaneous fat deposition in pigs is affected by lipase activity, so it is important to test the lipase activity of dorsal subcutaneous fat to study the impact of acorn diet on lipid metabolism in Yuxi black pigs. ACC represents a pivotal enzyme within the metabolic pathway responsible for the synthesis of fatty acids de novo. ACC can catalyze the carboxylation of acetyl-CoA to form malonyl-CoA, which is an intermediary in the de novo synthesis of fatty acids and plays a pivotal role in the regulation of fat deposition [20,21]. When ACC activity increases, it will promote lipid metabolism and fatty acid synthesis in animals [22]. Previous studies found that polyphenol-rich apple extracts can reduce ACC activity and inhibit lipid synthesis, which are in alignment with the results of the present study [23]. MDH is an important oxidoreductase in the TCA cycle. It has the capacity to catalyze the reversible conversion between malic acid and oxaloacetic acid in the citric acid cycle [24]. Oxaloacetic acid connects many important metabolic pathways in organisms. As a multi-substrate enzyme, the activity of MDH directly reflects the strength of fat synthesis [25]. Lee’s study found that MDH enzyme activity in Hampshire pigs with strong fat deposition capacity was higher than that in Duroc pigs with weak fat deposition capacity [26]. These studies suggest that acorns may inhibit fat deposition by reducing MDH activity. LPL is mainly synthesized by fat cells and discharged into the surface of fat cells or capillaries, where it can combine with triglyceride-rich lipoproteins in the circulatory system, hydrolyze triglycerides, release fatty acids for tissue absorption, and play a pivotal regulatory function in FAs’ transport and energy metabolism of fat cells [27]. Chang’s study found that the backfat thickness of fattening pigs decreased after the supplementation of fulvic acid in the diet, which may be caused by the increase in HSL activity and the decrease in LPL activity [28]. Meanwhile, Chen’s study found that the decrease in triglyceride content in the liver of weaned piglets would lead to a decrease in LPL enzyme activity [29]. The results indicated that acorns may reduce subcutaneous fat deposition by inhibiting LPL activity. In this study, this is consistent with the Tejeda study that found a reduced lipogenic enzyme activity in Iberian pigs fed acorns and grasses [19]. The results indicated that feeding the 30% acorn diet could inhibit subcutaneous fat deposition by inhibiting the lipase activities of ACC, MDH, and LPL in dorsal subcutaneous fat.

### 4.3. Analysis of Transcriptome Data Results

Dorsal subcutaneous fat deposition is closely related to backfat thickness. Consequently, an in-depth analysis of the molecular regulatory mechanisms governing the influence of the acorn diet on the development of dorsal subcutaneous fat via RNA-seq is of paramount importance for the advancement of pork quality. After removing low-quality reads, between 97.97% and 98.22% of the sequence reads obtained were mapped to the porcine reference genome for each sample. This represents a favorable outcome with regard to the analysis of RNA-seq data. A comparison of the gene expression profiles of dorsal subcutaneous fat in the two groups revealed that the overall number of genes expressed remained statistically similar between the two groups (CON = 13,815, AEG = 13,810), with 13,781 common genes identified as being expressed in both groups. This indicates that the majority of the dorsal subcutaneous fat transcriptome is preserved between the two animal groups. In addition, the GO enrichment analysis demonstrated that DEGs were significantly enriched in a number of pathways related to lipid metabolism, including the lipid metabolism process and lipid catabolic process, FA catabolic process, and FA β-oxidation. Previous studies through RNA-seq found that pigs with higher and lower backfat thicknesses may be attributed to variations in the regulatory mechanisms governing lipid metabolism, fatty acid synthesis, and lipid transport [30]. Furthermore, Gong, through RNA-seq, found that the DEGs of Tibetan pigs and Yorkshire pigs with different lipid deposition abilities were significantly enriched in lipid metabolism and lipid biosynthesis [31]. Consistent with the results of this study, feeding the 30% acorn diet can inhibit dorsal subcutaneous fat deposition by regulating the pathways related to lipid metabolism.

RNA-seq was conducted on dorsal subcutaneous fat samples, and 826 DEGs were screened in the present study, with 505 up-regulated and 321 down-regulated. These DEGs were classified to screen out key genes that regulate lipid deposition. These genes (*LEP*, *CHPT1*, *UCP3*, *ACOX1*, *SCD5*, *ACAA1*) reduce the ability of dorsal subcutaneous fat deposition by inhibiting fat synthesis or promoting lipolysis.

Leptin (*LEP*), also known as an anti-obesity factor, is mainly encoded by obesity genes and secreted by white fat cells [32]. Increased LEP expression can enhance the oxidation of FAs in tissues, improve the degradation of FAs in tissues, and ultimately achieve the purpose of reducing fat [33]. Some studies found that LEP can significantly improve the activity of the lipolysis enzymes HSL and ATGL [34,35]. In addition, LEP can down-regulate the SCD1 expression level, a gene associated with lipid metabolism, accelerate fatty acid hydrolysis, and inhibit fat deposition [36]. A large number of experiments proved that LEP can inhibit fat synthesis, promote fat decomposition, and improve the lipolysis of human and mouse fat cells [37]. The findings of the investigation demonstrated that the expression level of LEP in the AEG group was up-regulated. We hypothesized that this may be due to the up-regulation of LEP expression inhibiting FA synthesis and accelerating FA degradation, resulting in the inhibition of subcutaneous fat deposition. Choline phosphotransferase 1 (CHPT1), also known as CPT1 and CPT, is involved in the final step of the de novo synthesis of phosphatidylcholine, which is involved in the connection of two substrates, cytidine diphosphate choline (CDP-choline) and diglycerol ester [38]. The CHPT1 gene plays a role in fat deposition, facilitating the transfer of a portion of the phosphocholine from CDP-choline to lipids. The CHPT1 gene is involved in fat deposition, transferring part of the phosphocholine from CDP-choline to lipid-producing feedstock to produce phosphatidylcholine. Diglycerol is the raw material of triglyceride, and CHPT1 can consume diglycerol to inhibit fat deposition. The expression of CHPT1 was observed to vary between different age groups in the dorsal subcutaneous fat of pigs, with a negative correlation identified between this expression and the backfat thickness [31]. Our study found that the expression level of *CHPT1* was increased in the AEG group. We speculated that the up-regulation of *CHPT1* may promote the production of phosphatidylcholine, reduce cholesterol and triglyceride, and achieve the effect of inhibiting subcutaneous fat deposition.

Uncoupling protein 3 (*UCP3*) is an important transport protein in the inner mitochondrial membrane and is mainly distributed in the inner mitochondrial membrane of skeletal muscle. *UCP3* is a pivotal mitochondrial membrane protein involved in the transportation of essential molecules within skeletal muscle. The protein is able to facilitate the re-entry of protons driven out of the oxidative respiratory chain into mitochondria, causing the uncoupling of oxidative phosphorylation, thereby allowing energy to bypass ATP synthesis and be released as heat [39]. *UCP3* is closely related to the body’s energy balance and lipid metabolism. Previous studies found that a high-fat diet induces the up-regulation of *UCP3* expression levels in skeletal muscle, promotes the oxidation of fatty acids in skeletal muscle, and limits skeletal muscle’s efficiency in sugar utilization [40]. Duan’s study found that β-hydroxy-β-methyl butyrate can increase the expression levels of HSL and *UCP3* in cells, which in turn promotes the lipolysis of 3T3-L1 fat cells [41]. In this study, we found that *UCP3* expression was up-regulated in the AEG group, which may promote fatty acid oxidation and lipolysis to reduce dorsal subcutaneous fat deposition. Acyl-CoA oxidase 1 (*ACOX1*) is widely present in eukaryotic cells and is highly conserved. *ACOX1* is the rate-limiting enzyme for FA β-oxidation by oxidizing long-chain fatty acids to produce acyl-CoA, which in turn participates in the tricarboxylic acid cycle to generate energy. *ACOX1* plays a catalytic role in this process, promoting the oxidation of fatty acids [42]. Zhang’s research found that *ACOX1* defects lead to lipid accumulation and the excessive oxidation of polyunsaturated fatty acids (PUFAs) [43]. Chi found that ulva oligosaccharides were able to regulate the expression level of the lipid metabolism-related gene *ACOX1*, thus reducing blood lipids and liver lipid accumulation in mice [44]. The present study revealed that the expression level of ACOX1 in the AEG group is increased, which may promote the fatty acid β-oxidation of Yuxi black pigs and then regulate the dorsal subcutaneous fat deposition.

Stearoyl-CoA desaturase 5 (*SCD5*) is a key rate-limiting enzyme that facilitates the transformation of SFAs to MUFA, and its synthesis of MUFAs is a major substrate for the production of a variety of lipids, including triglycerides, cholesterol esters, and phospholipids [45]. Previous studies found that inhibiting SCD promotes the oxidation of FAs in the body [46]. When the body is deficient in SCD, it inhibits fatty acid synthesis and thus affects lipid metabolism [47]. It was postulated that the down-regulation of SCD5 expression in the dorsal subcutaneous fat of the AEG group not only inhibited FA synthesis but also promoted FA oxidation, thus attenuating the ability of dorsal subcutaneous fat to be deposited. Acetyl-CoA acyltransferase 1 (ACAA1) is a sulfurylase of the metabolizing enzyme acyl-CoA superfamily, also known as peroxisomal 3-ketoacyl CoA sulfurylase. ACAA1 plays a pivotal role in regulating FA β-oxidation within peroxisomes. It facilitates the cleavage of 3-ketoacyl-CoA, which results from the formation of acetyl-CoA and acyl-CoA, which are integral to the elongation and degradation of fatty acids [48]. The ACAA1 gene is situated at a subsequent point in the PPAR signaling pathway. PPAR enzymes exert tight control over the synthesis and transport of fatty acids. They catalyze the formation of esterified cholesterol from free cholesterol and long-chain FAs and perform the pivotal function in FA metabolism [49]. It was found that reduction in ACAA1 up-regulates the expression of the lipogenic genes PPARγ and C/EBPα in sheep preadipocytes, whereas the overexpression of ACAA1 inhibits PPARγ and C/EBPα expression, suppresses adipogenesis, and reduces lipid accumulation and triglyceride content [50]. We speculated that the up-regulation of ACAA1 expression in the AEG group may accelerate the oxidation of FAs in the PPAR signaling pathway, thereby inhibiting the dorsal subcutaneous fat deposition, but the specific mechanism still needs to be further studied.

In conclusion, acorns, as a wild plant rich in polyphenols, can be used as 30% acorns in a basal diet, which can have a positive effect on improving the subcutaneous fat deposition and reducing backfat thickness of Yuxi black pigs by regulating the expression of genes related to lipid metabolism, fat synthesis, and the fat decomposition pathway. In addition to the above differentially expressed genes that affect lipid metabolism by regulating fat synthesis and lipolysis, this study additionally revealed that *ADIPOQ*, *ACADL*, *ACOT4,* and *ACSL1* may play a role in the regulation of dorsal subcutaneous fat deposition of Yuxi black pigs. Nevertheless, further investigation is required to elucidate the precise relationship and the underlying mechanisms involved in lipid metabolism.

## 5. Conclusions

In conclusion, feeding a 30% acorn diet can reduce the lipase activities, regulate the expression levels of DEGs involved in pathways related to lipid metabolism, inhibit dorsal subcutaneous fat deposition, and reduce backfat thickness of Yuxi black pigs.

## Figures and Tables

**Table 1:** Diet composition and nutrient levels.

Item	Content	
	CON	AEG
Ingredient, %		
Corn	65.5	44.9
Soybean meal	3.0	5.9
Wheat bran	22.2	7.5
Walnut dregs	5.3	7.7
Acorn	0	30.0
Premix	4.0	4.0
Total	100	100
Nutrient levels		
ME, MJ·kg^−1^	11.8	11.4
CP, %	11.9	11.5
Lys, %	0.5	0.5
Cys, %	0.3	0.2
Met + Cys, %	0.5	0.4
Protein energy ratio, g·MJ^−1^	10.1	10.1

**Table 2:** Primer sequences.

Gene	Accession Number	Primer Sequences, 5′-3′
*ACAA1*	XM_003132103.4	F: ACGATGACAAGGGCAATGAGAAGAG R: ACAGGGATGGCATAGGCAGGTC
*ACADL*	NM_213897.1	F: TTACAAATCGTGAAGCTCGCTCTCC R: CACCGTCTGTATATGTGCCACTGTC
*ADIPOQ*	EF601160.1	F: TGTCTACCGTTCAGCATTCAGTGTG R: GAAGGAAGCCTGTGAAGATGGAGTC
*SCD5*	NM_001114278.1	F: ATCCTCCGCTACACCGTCTCAC R: CAGCCAGCACATGAAGTCAATGAAC
*GAPDH*	NM_001206359.1	F: CAAGGCTGTGGGCAAGGTCATC R: AAGTGGTCGTTGAGGGCAATGC
*ACOX1*	NM_001101028.1	F: GTATGCCCAGGTGAAGCCAGATG R: ATTAGCCGTCCAGGATGTGAAAGC
*UCP3*	NM_214049.1	F: CCATCGCCAGGGAGGAAGGG R: TGTCCGTGAGCAGGTGGTAGTC

**Table 3:** Quality statistics of transcriptome sequencing data.

Sample	Clean Reads	Clean Bases	Useful Reads%	Useful Bases%	GC Content%	Q30
BZ_CON1	44,888,886	6,737,128,958	98.01	97.42	46.48	94.94
BZ_CON2	45,363,754	6,816,335,257	98.06	97.58	47.17	95.09
BZ_CON3	46,014,420	6,884,658,949	98.16	97.27	45.83	95.31
BZ_AEG1	41,436,780	6,209,061,499	97.97	97.22	46.02	95.06
BZ_AEG2	47,571,278	7,129,574,508	98.14	97.41	47.16	95.01
BZ_AEG3	53,530,398	8,042,326,168	98.22	97.72	47.04	95.23

BZ_CON was the dorsal subcutaneous fat of the control group; BZ_AEG was the dorsal subcutaneous fat of the AEG group.

**Table 4:** Statistical analyses of the sequencing data of samples and the sequence alignment results of selected reference genomes.

Sample	Total Reads	Mapped Reads	Multiple Mapped	Uniquely Mapped	Exon Mapped	Intergene Mapped
BZ_CON1	44,888,886	43,826,402 (97.63%)	1,043,665 (2.38%)	42,782,737 (97.62%)	34,555,175 (84.95%)	2,107,313 (4.93%)
BZ_CON2	45,363,754	44,421,001 (97.92%)	1,088,487 (2.45%)	43,332,514 (97.55%)	36,293,893 (87.42%)	1,813,785 (4.19%)
BZ_CON3	46,014,420	44,936,991 (97.66%)	1,177,070 (2.62%)	43,759,921 (97.38%)	35,889,451 (86.09%)	2,072,345 (4.74%)
BZ_AEG1	41,436,780	40,508,660 (97.76%)	972,344 (2.40%)	39,536,316 (97.60%)	33,464,234 (88.57%)	1,755,463 (4.44%)
BZ_AEG2	47,571,278	46,502,896 (97.75%)	1,159,622 (2.49%)	45,343,274 (97.51%)	37,684,542 (86.75%)	1,900,984 (4.19%)
BZ_AEG3	53,530,398	52,438,782 (97.96%)	1,331,336 (2.54%)	51,107,446 (97.46%)	43,247,481 (88.23%)	2,088,170 (4.09%)

BZ_CON was the dorsal subcutaneous fat of the control group; BZ_AEG was the dorsal subcutaneous fat of the AEG group.

**Table 5:** Differentially expressed gene that influence lipid metabolism.

Gene	Gene Name	Gene Description	Log2FC	*p*-Value
ENSSSCG00000040464	*LEP*	leptin	2.146658349	0.037113913
ENSSSCG00000000864	*CHPT1*	choline phosphotransferase 1	1.10832771	0.000429612
ENSSSCG00000014834	*UCP3*	uncoupling protein 3	3.804562568	1.73 × 10^−19^
ENSSSCG00000017198	*ACOX1*	acyl-CoA oxidase 1	1.274871269	3.53 × 10^−5^
ENSSSCG00000009245	*SCD5*	stearoyl-CoA desaturase 5	−1.55683637	0.013021827
ENSSSCG00000011250	*ACAA1*	acetyl-CoA acyltransferase 1	2.387704936	3.05 × 10^−8^
ENSSSCG00000039103	*ADIPOQ*	adiponectin	2.767298331	1.20 × 10^−9^
ENSSSCG00000016156	*ACADL*	acyl-CoA dehydrogenase long chain	1.175895382	0.000321714
ENSSSCG00000015784	*ACSL1*	acyl-CoA synthetase long chain family member 1	1.851766159	0.000145487
ENSSSCG00000002349	*ACOT4*	acyl-CoA thioesterase 4	0.746617108	0.014478849

## Data Availability

Should any interested parties require access to the datasets generated and/or analyzed during the course of the present study, they are invited to contact the corresponding author to make a reasonable request.
